# Environmental risk assessment of pesticides in the River Madre de Dios, Costa Rica using PERPEST, SSD, and msPAF models

**DOI:** 10.1007/s11356-016-7375-9

**Published:** 2016-09-12

**Authors:** Robert A. Rämö, Paul J. van den Brink, Clemens Ruepert, Luisa E. Castillo, Jonas S. Gunnarsson

**Affiliations:** 1 Department of Ecology, Environment and Plant Sciences (DEEP), Stockholm University, 106 91 Stockholm, Sweden; 2 Alterra, Wageningen University and Research Centre, Wageningen, The Netherlands; 3 Department of Aquatic Ecology and Water Quality Management, Wageningen University, Wageningen, The Netherlands; 4 Central American Institute for Studies on Toxic Substances (IRET), Universidad Nacional, Heredia, Costa Rica

**Keywords:** Aquatic pollution, Agricultural runoff, Mixture toxicity, ERA, Central America, Tropical ecotoxicity

## Abstract

This study assesses the ecological risks (ERA) of pesticides to aquatic organisms in the River Madre de Dios (RMD), which receives surface runoff water from banana, pineapple, and rice plantations on the Caribbean coast of Costa Rica. Water samples collected over 2 years at five sites in the RMD revealed a total of 26 pesticides. Their toxicity risk to aquatic organisms was assessed using three recent ERA models. (1) The PERPEST model showed a high probability (>50 %) of clear toxic effects of pesticide mixtures on algae, macrophytes, zooplankton, macroinvertebrates, and community metabolism and a low probability (<50 %) of clear effects on fish. (2) Species sensitivity distributions (SSD) showed a moderate to high risk of three herbicides: ametryn, bromacil, diuron and four insecticides: carbaryl, diazinon, ethoprophos, terbufos. (3) The multi-substance potentially affected fraction (msPAF) model showed results consistent with PERPEST: high risk to algae (maximum msPAF: 73 %), aquatic plants (61 %), and arthropods (25 %) and low risk to fish (0.2 %) from pesticide mixtures. The pesticides posing the highest risks according to msPAF and that should be substituted with less toxic substances were the herbicides ametryn, diuron, the insecticides carbaryl, chlorpyrifos, diazinon, ethoprophos, and the fungicide difenoconazole. Ecological risks were highest near the plantations and decreased progressively further downstream. The risk to fish was found to be relatively low in these models, but water samples were not collected during fish kill events and some highly toxic pesticides known to be used were not analyzed for in this study. Further sampling and analysis of water samples is needed to determine toxicity risks to fish during peaks of pesticide mixture concentrations. The msPAF model, which estimates the ecological risks of mixtures based on their toxic modes of action, was found to be the most suitable model to assess toxicity risks to aquatic organisms in the RMD. The PERPEST model was found to be a strong tool for screening risk assessments. The SSD approach is useful in deriving water quality criteria for specific pesticides. This study, through the application of three ERA models, clearly shows that pesticides used in plantations within the RMD watershed are expected to have severe adverse effects on most groups of aquatic organisms and that actions are urgently needed to reduce pesticide pollution in this high biodiversity ecosystem.

## Introduction

Costa Rica has among the highest biodiversity on earth and is known for its nature conservation efforts and eco-tourism. It is also a major agro-economy and among the world’s largest producers of banana and pineapple (FAO 2014; FAOSTAT 2016). The banana production is achieved through large monocultures located in the tropical Caribbean lowlands (CORBANA 2013). Pesticide use in these plantations is intensive, with 49 kg active ingredients (a.i.) per hectare and year applied in banana plantations and 25 kg a.i. per hectare and year applied in pineapple plantations (Bravo et al. 2013). These plantations are rain-fed, but heavy rainfall in the Caribbean region (3.2 m average precipitation per year) requires that plantations drain excess rainwater through drainage canals (Grant et al. 2013), leading to discharges of untreated surface runoff water into rivers downstream these plantations.

**Fig. 1:**
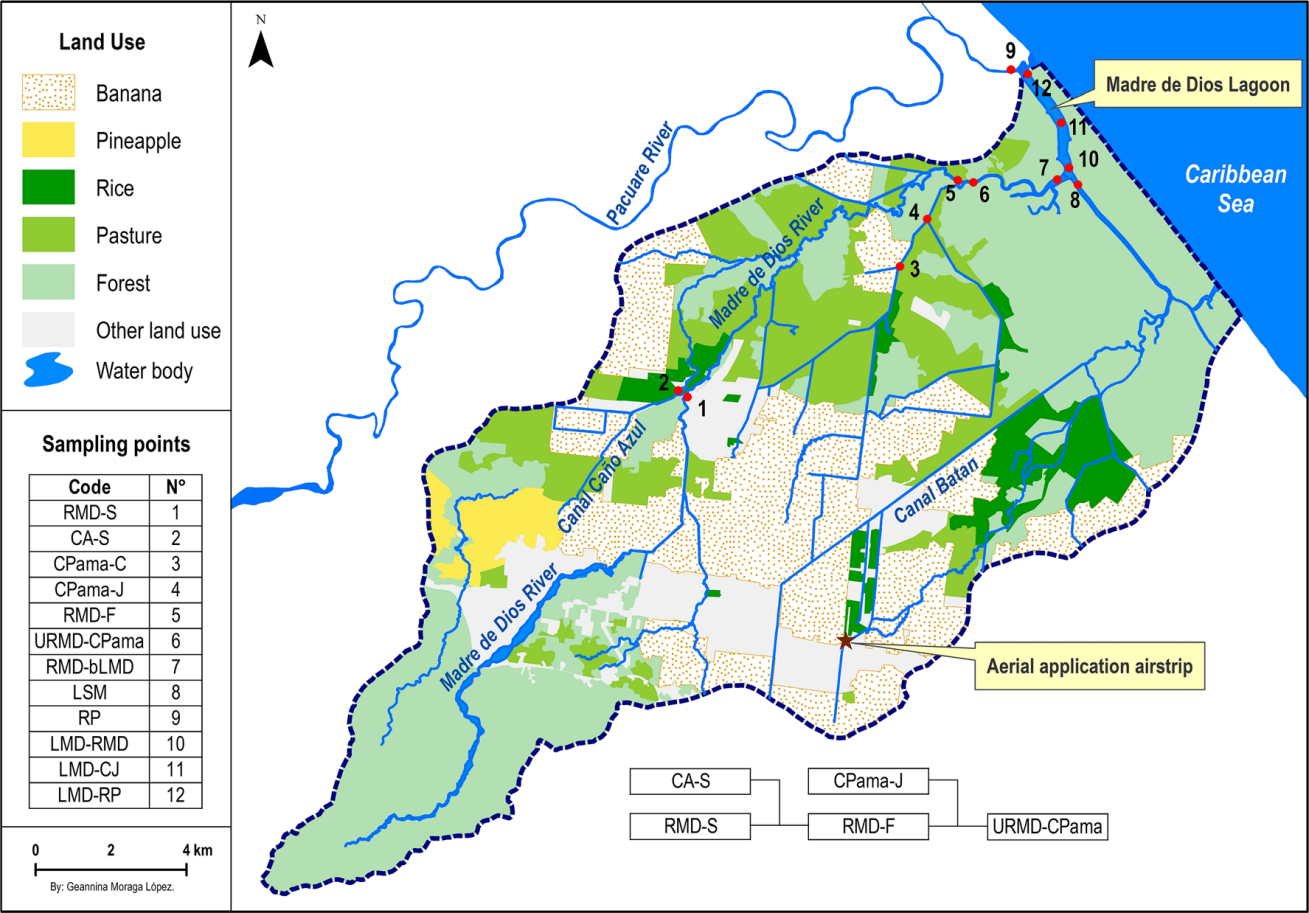
Land use map of the River Madre de Dios watershed. Banana accounts for the largest portion of agricultural land use, followed by rice and pineapple. *Center bottom*: schematic diagram of the five sampling sites with water flow direction from *left* to *right*. The sites CA-S and CPama-J are located in tributaries. Map by Geannina Moraga, Centre de GIS, IRET, UNA, Heredia, Costa Rica

Pesticide contamination of Costa Rican wildlife has previously been reported (de la Cruz et al. 2014; Klemens et al. 2003) and both acute and chronic effects have been observed in aquatic ecosystems downstream plantations (Castillo et al. 2006; Castillo et al. 1997; Echeverria-Saenz et al. 2012). The River Madre de Dios (RMD) watershed (10.1921°N 83.2953°V) consists of a river and coastal lagoon in the province of Limón on the Caribbean coast of Costa Rica. This watershed has a high biodiversity and provides local residents with income from fishery and ecotourism, but it also hosts large monocultures of banana, pineapple, and rice (Fig. 1). Frequent fish kills have been reported in the RMD since 2004 (18 observed events between 2007 and 2009), and these have been suggested to be caused by pesticide runoff (CGR 2013; Diepens et al. 2014).

Studies are needed to find the causes of these fish kills and to characterize the toxicity risks of pesticide pollution in the RMD. However, there is a lack of knowledge on how to assess and mitigate risks of chemicals in tropical countries: also, many Central American countries do not have or do not enforce environmental regulations. The pesticide registration process in Costa Rica consists of a simple risk quotient approach based on US EPA guidelines, where the aquatic toxicity evaluation consists of acute and chronic tests on three standard species (MINAE 2011).

The relevance of using standard test species from temperate systems to predict toxicity risks in tropical systems may be questioned as tropical and temperate systems differ in several ways that may affect the risks of pesticides, e.g., soil and sediment types (affecting sorption and degradation rates of chemicals), temperature, sunlight, and pH (Sanchez-Bayo and Hyne 2011). Tropical ecosystems are often thought to contain more sensitive species and would therefore be more vulnerable to pesticides than temperate ones. Some recent studies have explored these differences, e.g., Maltby et al. (2005) found no influence of geographical distribution on species sensitivity distributions (SSDs) of insecticides, and Daam and Van den Brink (2010) found no systematic difference in chlorpyrifos degradation and toxicity between temperate and tropical systems. Rico et al. (2011) found no statistical difference in toxicity of the insecticide malathion on tropical freshwater (Amazonian) fish and invertebrates compared to temperate fish, but did find that tropical species were more robust to the fungicide carbendazim. They concluded that tropical species are protected when using threshold values (HC_5_) derived from SSD on temperate species, provided that sufficient representative species are used in the SSD (Rico et al. 2011). On the other hand, Kwok et al. (2007) found that tropical species may be more sensitive to some pesticides, e.g., chlorpyrifos, based on toxicity studies on a range of substances. In a recent toxicity study, Diepens et al. (2014) compared the temperate cladoceran *Daphnia magna* to its tropical counterpart *Daphnia ambigua* and found that *D. ambigua* is more sensitive than *D. magna*, the standard species used for aquatic toxicity assessment in Costa Rica, which implies that the current pesticide registration process in Costa Rica may be underprotective. These studies highlight that further ecotoxicological research is needed in tropical ecosystems, including studies on tropical endemic species, but also suggest that species in tropical and temperate regions do not appear to have fundamentally different responses to toxic substances.

Many different methods have been proposed to derive toxicity risk values for pesticides. The species sensitivity distribution (SSD) describes the variation in species’ sensitivity for a particular toxic substance by fitting pre-existing toxicity data for relevant species to an assumed (often log-normal) distribution (Aldenberg and Jaworska 2000). The SSD concept can be used in risk assessment to calculate the potentially affected fraction (PAF) of species from exposure to an environmental contaminant and is also used to derive environmental quality standards (EQS): concentration thresholds under which a fraction of species is protected from toxic effects, e.g., a 95 % protection level from HC_5_, the Hazard Concentration for 5 % of species (Kooijman 1987). SSD is a widely recognized method for toxicity assessments of single substances and for the development of water quality standards for environmental pollutants. It is a standard concept used in the EU, Canada, and the USA (CCME 2007; EFSA 2013; USEPA 2000) but has not yet been implemented in Costa Rican guidelines. SSD is often recommended as a tool for assessing the toxicity risks of individual substances, but mixtures of substances more often occur in the environment and risk assessments therefore need to account for the joint toxicity of mixtures (Suter et al. 2002).

The multi-substance PAF (msPAF) model is designed to assess the toxicity risk of mixtures using the SSD principles. The msPAF model applies concentration addition (CA) to calculate a single risk value for substances that have a shared toxic mode of action (TMoA) and then applies response addition (RA) to sum the toxicity risks of each TMoA. The result is a msPAF value that describes the potentially affected fraction of species from exposure to a complex mixture (de Zwart and Posthuma 2005; Traas et al. 2002). The CA and RA models underpinning the msPAF model have been separately experimentally validated, where observed effects of known mixtures match the predicted effects from the respective models (Altenburger et al. 2000; Faust et al. 2003). The SPEAR_pesticides_ bioindicator, a trait-based ecological index for steam invertebrates, also correlates well with predictions made by the msPAF model (Smetanova et al. 2014).

The msPAF approach is applicable provided that each component of a mixture has a known TMoA. There is, however, no consensus on what constitutes a distinct mode or mechanism of action (Lambert and Lipscomb 2007) and it has been advised that experimental validation of TMoA is necessary for the regulatory use of multiple TMoA (and consequently, of RA) in mixture assessments (Backhaus et al. 2013). The CA model is therefore often used as the default mixture model (and has been called a “General Solution”) but overestimates the risk of mixtures with multiple TMoA when compared to the mixed model approach which applies both CA and RA. The present study applies CA and RA models in msPAF for the purpose of ERA without experimental validation of the TMoA.

The predict the ecological risks of pesticides (PERPEST) model has been developed for risk assessment of both single pesticides and pesticide mixtures. It applies a case-based reasoning process to compare a current case of pesticide pollution to a database (“the case base”) containing toxicity data from pesticide mesocosm experiments with known outcomes (Van den Brink et al. 2002). The model compares environmental concentrations of pesticides to previous results from the case base to estimate probabilities of toxic effects on several species (e.g., algae, macrophytes, zooplankton, macroinvertebrates, fish, and tadpoles) and on community metabolism (i.e., respiration, primary production). Thus, the PERPEST model accounts not only for direct effects on species but also for indirect effects and interactions among species (i.e., prey-predator effects) that are observed in mesocosms but not in the single species tests used in SSD and msPAF models.

In the present study, we applied the three models for environmental risk assessment (ERA) presented above to assess the toxicity risks of pesticides in the RMD: (1) the PERPEST model, (2) the SSD method, and (3) the msPAF method. Results from the three models are compared and the advantages and drawbacks of each model are discussed. Recommendations are made for further ERA in tropical aquatic ecosystems.

## Methods

### Sampling sites

The sampling sites chosen for this study are part of a larger sampling effort in the RMD comprising 12 sites in total. Five sites were assessed in this study and are labeled 1, 2, 4, 5, and 6 on the map (Fig. 1). Three of the study sites are located in the river (RMD) and two sites are located in tributaries that receive untreated surface runoff water from agricultural lands. These sites were chosen to represent an exposure gradient from plantations towards the recipient coastal lagoon. These five study sites are as follows: (1) RMD-S, located upstream of most plantation discharges, (2) CA-S, located in the Caño Azul tributary that receives surface runoff from pineapple and banana plantations, (3) RMD-F, located further downstream of RMD-S and CA-S, (4) CPama-J, located in the Canal Pama tributary that receives surface runoff from mainly banana plantations, and (5) URMD-CPama, located further downstream of RMD-F and CPama-J.

### Water sampling and pesticide analysis

We collected 68 surface water samples on 15 sampling occasions at the study sites over a 2-year period (2011–2012). Water samples were collected via boat by inserting pre-washed 2-L brown glass bottles into the water. The bottles were transported in cooled ice boxes to the laboratory LAREP, UNA, Heredia, Costa Rica and stored at 4–6 °C until analyses. The water samples were extracted on solid phase extraction columns and the extracts were analyzed by GC-MS (Agilent 7890A GC and 5975C MS, Palo Alto, USA) for non-polar pesticides and HPLC with diode array detection for polar pesticides. LC-PDA analyses were performed using a Shimadzu HPLC LC-10AD with an SPD-M10A diode array detector (Shimadzu, Kyoto, Japan). The chromatographic column was a LiChroCART HPLC RP-18e column (125 mm × 3 mm × 5 μm particle size, Merck, Germany). Fifty microliters of extracts was analyzed. The mobile phase consisted of 20-mM sodium acetate in ultra-pure water/methanol 56:44 (solvent A) and methanol (solvent B). Identification was performed using retention time and the UV spectra of the pesticides included in the analysis. Pesticide residues analyzed by GC-MS were identified using the Chemstation software and the NIST05 Mass Spectral Database, and concentrations were determined using external standards. A selection of 32 pesticides and pesticide metabolites were included in the analysis based on available external standards from pesticides reported in the pesticide registration process and in interviews with farmers and crop owners. Several groups of pesticides that may cause high risks were not analyzed, including pyrethroids, neonicotinoids, and some fungicides with high volumes of use in Costa Rica.

### Physico-chemical and toxicological properties of detected pesticides

Information on the chemical characteristics of pesticides detected in the field was collected from the literature, including chemical abstract service (CAS) registry number, common name, molecular weight, vapor pressure, Henry’s law constant, half degradation time in water (DT_50_), and octanol-water partition coefficient (*K*_ow_) from the Pesticide Properties Database (Lewis et al. [2016], available at sitem.herts.ac.uk/aeru/ppdb, last accessed on 2015-11-01). Literature toxicity data was obtained from the U.S. EPA Ecotox database (USEPA 2016) and the E-toxBase (De Zwart 2002). Selected test organisms were algae (microalgae, cyanobacteria), aquatic plants, arthropods (aquatic insects, crustaceans), and fish. Only freshwater laboratory tests with suitable test conditions were used to reduce inconsistencies from different experimental systems. As lethal effects in fish are observed in the field, we collected median effect concentrations (EC_50_) on mortality for all species, on immobility in mobile species, on inhibition of cell division in algae, and on growth inhibition in aquatic plants. Exposure times were 1–7 days for algae, aquatic insects, and crustaceans; 2–21 days for fish; 2–28 days for aquatic plants (Maltby et al. 2009). It should, however, be noted that 98.3 % of toxicity tests used for aquatic insects and crustaceans had exposure times between 1 and 4 days. Mean effect concentrations were calculated for each species-pesticide combination and were used to model species sensitivity with equal weight of each included species, i.e., any bias towards often tested species were removed by using one toxicity value per species.

### Toxic modes of action

The present study uses classifications provided in the *Compendium of Pesticide Common Names* (Alan Wood, available at www.alanwood.net/pesticides; last accessed on 2015-08-25) to categorize pesticides by their TMoA, following recommendations of De Zwart and Posthuma (2005). This database identifies molecular classes of pesticides and is similar to an approach using molecular classes used by Gregorio et al. (2012). Similarly, De Zwart (2002) reported 68 TMoA identified either by molecular classes or QSAR, an approach that has since been expanded and applied to the management of European river basins (de Zwart et al. 2009). However, other sources of TMoA information are available: Jesenska et al. (2013) used classifications based on specific binding sites of herbicides, e.g., mechanisms, rather than modes, of action and Altenburger et al. (2013) took a similar approach using the classifications of the insecticide, fungicide, and herbicide resistance action committees. These committees catalogue modes of action to develop pesticide resistance management strategies, and it could be assumed that pesticides for which cross-resistance is developed share a common mode or mechanism of action. There are thus several sources available that identify TMoA for pesticides, and there is a need for further studies to identify the most suitable source of information for use in mixture toxicity modeling.

### PERPEST

The PERPEST software (Van den Brink et al. [2006b]; Van den Brink et al. [2002]; version 4.0.0) was used to predict the probability of effects from pesticide mixtures in the RMD (www.perpest.wur.nl). Probabilities of no effects, slight effects, and clear effects were calculated for (1) algae and macrophytes, (2) zooplankton, (3) macroinvertebrates, (4) fish and tadpoles, and (5) community metabolism. Pesticides not currently available in the PERPEST case base were added using physico-chemical properties obtained from the literature and median hazard concentrations (HC_50_) calculated in the present study. The PERPEST program was used with default settings, except that cases from the case base were weighted using TMoA and toxic units (TU) and selected using TMoA and nearby TU. The results are presented as low or high probability of clear effects, where a low probability is defined as below 50 % and signifies a low risk, and a high probability is 50 % or higher and signifies a high risk.

### Species sensitivity distributions

SSDs were generated using collected toxicity data with equal weight of species. Fish and arthropod species were used to generate insecticide SSDs, algae and aquatic plant species for herbicide SSDs, and all taxonomic groups mentioned above were used for fungicide SSDs. The ETX software (Van Vlaardingen et al. (2004); version 2.1) was used to generate SSDs with a confidence limit-based estimator, the best performing method to fit a SSD (Hickey and Craig 2012). Log-normality of species toxicity data was assessed using the Anderson-Darling test in ETX. Failure to meet the 5 % critical level resulted in rejecting the SSD and generating SSDs for minor taxonomic groups, until normality was met. The most sensitive group (based on HC_5_) to meet normality criteria was used. Pesticides with no log-normal distribution or insufficient sample size (<6 species) were not assessed with SSD. The hazard concentrations for 5 % of species (HC_5_) and 50 % of species (HC_50_) were extracted from the SSD of each pesticide, following the calculation of a potentially affected fraction of species (PAF) using the maximum measured environmental concentration of the pesticide in each site. The results are interpreted as low risk under 1 % PAF, as moderate risk above 1 % PAF, and as high risk above 5 % PAF, corresponding to the commonly used HC_5_ benchmark.

### Multi-substance PAF

Six species groups were assessed using msPAF. First, (1) primary producers and (2) fish and arthropods were used to maximize sample sizes and for comparison to the SSD results, then distinct taxonomic groups were selected to study specific effects on (3) algae, (4) aquatic plants, (5) fish, and (6) arthropods. The method used to calculate msPAF follows De Zwart and Posthuma (2005) with modifications. A hazard unit (HU) was calculated for each species group-pesticide combination as the geometric mean of literature toxicity data (similar to the HC_50_). These HU units were used to scale toxicity data and measured environmental concentrations (MEC) of pesticides to dimensionless HU values to adjust for differences in the potency of pesticides. Mean (α) and standard deviation (σ) of log toxicity data (expressed in HU units) were calculated for each pesticide using equal weight of species for α but taking intra-species variance into account for σ. Each pesticide was assigned a TMoA based on molecular activity. These TMoA were also considered for non-target species, following de Zwart et al. (2009). The TMoA groups were evaluated using calculated SSD slopes, where pesticides with slopes (σ) deviating more than ±10 % from others were assigned to a separate TMoA. The CA model was used to calculate a PAF value for each TMoA (msPAF_CA_) in a sample using the Microsoft Excel© function (1).

1 $$ \mathrm{NORM}.\mathrm{DIST}\left({MEC}_{\mathrm{TMoA}},\upalpha, \upsigma, 1\right) $$

Where MEC_TMoA_ is the total MEC of pesticides in the TMoA, *ɑ* is the average *ɑ*_*i*_ for pesticides *i* = 1 to i = *n* in the TMoA, and σ is the average σ_*i*_ for pesticides *i* = 1 to *i* = *n* in the TMoA. After obtaining msPAF_CA_ for each TMoA, the total toxicity of a sample (msPAF_RA_) was calculated using the following formula for the RA model (2):

2 $$ {\mathrm{msPAF}}_{RA}=1-{\displaystyle {\prod}_{i=1}^n\left(1-{\mathrm{msPAF}}_{CA,i}\right)} $$

Pesticides with insufficient sample size (<4 species) were not assessed with msPAF. This minimum sample size was set following calculation of the effects of minimum sample size on pesticide coverage (and subsequent toxicity risks), where msPAF was modeled with a minimum sample size of either 2, 4, 6, or 10 species. This evaluation indicated that the number of assessable pesticides and resulting toxicity risks decrease with sample size, particularly above a minimum of 4 species (see discussion). The results are interpreted analogous to SSD, where low risks occur below 1 % PAF, moderate risk occur between 1 and 5 % PAF, and high risk occur above 5 % PAF.

## Results

### Measured environmental concentrations

There were 26 pesticides detected at the sampling sites: 13 fungicides, 7 herbicides, and 6 insecticides (Table 1). Detection frequencies varied from 1 to 48 occurrences per pesticide in a total of 68 samples. The herbicide diuron was the most commonly detected pesticide and was found in 62 % of samples. Water samples contained a median of 4 pesticides and a maximum of 16 pesticides. The median concentration of a pesticide was 0.13 μg/L (excluding non-detects), and the maximum was 24.0 μg/L for diuron (Table 1).

**Table 1:** Pesticide occurrences and measured environmental concentrations (MEC) in 68 samples at five sites in the RMD watershed (2011–2012)

CAS	Common name	Type	Analyzed samples (*n*)	Detections (*n*)	Avg. MEC (μg/L)	σ	Max. MEC (μg/L)
131860-33-8	Azoxystrobin	F	54	27	0.74	0.65	2.20
055179-31-2	Bitertanol	F	68	1	0.13	–	0.13
001897-45-6	Chlorothalonil	F	68	15	0.05	0.04	0.12
119446-68-3	Difenoconazole	F	68	12	0.36	0.22	1.00
135319-73-2	Epoxiconazole	F	68	37	0.30	0.19	0.78
035554-44-0	Imazalil	F	68	1	0.50	–	0.50
057837-19-1	Metalaxyl	F	67	12	0.06	0.04	0.14
088671-89-0	Myclobutanil	F	46	10	0.17	0.17	0.60
060207-90-7	Propiconazole	F	68	17	0.13	0.08	0.35
053112-28-0	Pyrimethanil	F	68	35	0.19	0.17	0.81
107534-96-3	Tebuconazole	F	58	7	0.27	0.21	0.60
000148-79-8	Thiabendazole	F	68	1	0.78	–	0.78
055219-65-3	Triadimenol	F	68	1	0.10	–	0.10
000834-12-8	Ametryn	H	68	37	0.75	3.27	20.0
000314-66-9	Bromacil	H	68	4	0.65	0.74	1.70
023184-66-9	Butachlor	H	40	1	0.04	–	0.04
000330-54-1	Diuron	H	67	42	0.90	3.68	24.0
051235-04-2	Hexazinone	H	68	6	0.14	0.11	0.33
042874-03-3	Oxyfluorfen	H	40	2	0.03	0.01	0.03
000886-50-0	Terbutryn	H	68	4	0.04	0.03	0.08
000063-25-2	Carbaryl	I	67	9	0.62	0.72	2.20
002921-88-2	Chlorpyrifos	I	68	19	0.04	0.03	0.15
000333-41-5	Diazinon	I	68	22	0.13	0.14	0.63
013194-48-4	Ethoprophos	I	68	28	0.22	0.32	1.56
022224-92-6	Fenamiphos	I	68	7	0.12	0.05	0.18
013071-79-9	Terbufos	I	68	1	0.05	–	0.05

The pesticide metabolites carbofuran phenol and terbufos sulfone and the chemicals deet and dichloroaniline were not included in the toxicity risk assessment

*F* fungicide, *H* herbicide, *I* insecticide

### PERPEST

The pre-compiled PERPEST case base contained mesocosm effect data for 3 of the herbicides and 4 of the insecticides detected in the RMD: the herbicides diuron, hexazinone, and terbutryn and the insecticides carbaryl, carbofuran, chlorpyrifos, and diazinon. The 19 remaining pesticides were added to the model using their physico-chemical properties and toxicity data from the literature (Table 2). The results show that clear effects are likely (>50 % maximum probability) to occur on community metabolism and on the species communities of algae and macrophytes, zooplankton, and macroinvertebrates (Table 3). Clear effects were, however, not likely to occur on fish and tadpoles in any sampling site (the maximum probability of clear effect on fish and tadpoles was 43 %).

**Table 2:** Pesticide properties entered into the PERPEST program for assessment of pesticide mixtures. Physico-chemical properties retrieved from the PPDB (Lewis et al. 2016). HC_50_ derived in this paper

CAS	Pesticide name	Type	Mode of action	Molecule group	Aquatic phase DT_50_ (d)	HC_50_ (μg/L)	Henry's law constant at 25°C (Pa m^3^ mol^-1^)	K_ow_ at 20 °C
131860-33-8	Azoxystrobin	F	Other fungicides	–	6.10	578.3	7.40E-09	316.0
055179-31-2	Bitertanol	F	Other fungicides	–	27.00	3582	2.60E-07	12,600
001897-45-6	Chlorothalonil	F	Other fungicides	–	0.10	92.19	2.50E-02	871.0
119446-68-3	Difenoconazole	F	Other fungicides	–	3.00	196.1	9.00E-07	22,900
135319-73-2	Epoxiconazole	F	Other fungicides	–	65.80	9900	4.71E-04	2000
035554-44-0	Imazalil	F	Other fungicides	–	7.80	2193	1.08E-04	363.0
057837-19-1	Metalaxyl	F	Other fungicides	–	56.00	105,968	1.60E-05	56.10
088671-89-0	Myclobutanil	F	Other fungicides	–	12.00	5177	4.33E-04	776.0
060207-90-1	Propiconazole	F	Other fungicides	–	6.00	3845	9.20E-05	5250
053112-28-0	Pyrimethanil	F	Other fungicides	–	16.50	15,786	7.42E-07	692.0
107534-96-3	Tebuconazole	F	Other fungicides	–	42.60	2139	1.00E-05	5010
000148-79-8	Thiabendazole	F	Other fungicides	–	1.60	10,720	3.70E-06	245.0
055219-65-3	Triadimenol	F	Other fungicides	–	53.00	28,191	3.50E-06	1510
000834-12-8	Ametryn	H	Photosynthesis inhibitor	Triazin(on)e	Stable	7.71	4.10E-04	426.6
000314-40-9	Bromacil	H	Photosynthesis inhibitor	–	Stable	28.45	1.50E-05	75.86
023184-66-9	Butachlor	H	Other herbicide	–	n/a	3431	3.74E-03	31,623
042874-03-3	Oxyfluorfen	H	Other herbicide	–	5.6	402.2	2.38E-02	72,444
013194-48-4	Ethoprophos	I	Acetylcholinesterase inhibitor	Organophosphate	20.00	342.2	1.35E-02	977.0
022224-92-6	Fenamiphos	I	Acetylcholinesterase inhibitor	Organophosphate	5.80	13.57	9.90E-05	2000
013071-79-9	Terbufos	I	Acetylcholinesterase inhibitor	Organophosphate	4.5	7.15	2.70E + 00	32,400

Aquatic phase DT50: *n/a* not available, *stable* stable compound in water: 999 entered into the PERPEST program; *DT*_*50*_ half-life degradation time; *HC50* hazard concentration for 50 % of species; *K*_*ow*_ octanol/water partition coefficient

*F* fungicide, *H* herbicide, *I* insecticide

**Table 3:** Average, standard deviation (in parenthesis), and maximum (bold text) probability of clear effect (%) derived from PERPEST for pesticide mixtures at each of the five study sites. Average number of analogous cases for predictions of each endpoint in the PERPEST case base

	Study sites										
Endpoint	RMD-S		CA-S		RMD-F		CPama-J		URMD-CPama		Analogous cases
Algae and macrophytes	–	–	42 (14)	**84**	34 (12)	**44**	26 (18)	**40**	32 (12)	**40**	11.4
Zooplankton	5 (9)	**18**	45 (16)	**79**	43 (17)	**61**	37 (21)	**59**	44 (16)	**56**	9.3
Macroinvertebrates	3 (5)	**11**	39 (15)	**73**	25 (18)	**56**	12 (12)	**38**	19 (17)	**44**	4.2
Fish and tadpoles	–	–	19 (11)	**43**	23 (3)	**25**	3 (7)	**15**	25 (7)	**32**	1.3
Community metabolism	–	–	34 (22)	**95**	18 (19)	**60**	6 (10)	**32**	13 (13)	**43**	9.1

Blank (–) indicates no result was obtained (no analogous cases or estimation out of bounds, i.e., near-zero risk)

The highest probabilities of clear effects to all endpoints was observed at the site CA-S, with a 95 % probability of clear effects on community metabolism, an 84 % probability on algae and macrophytes, a 79 % probability on zooplankton, a 73 % probability on macroinvertebrates, and a 43 % probability of clear effects on fish and tadpoles. The results also show high variance in the probability of clear effect within sites (Table 3), with coefficients of variance ranging from 0.13 to 2.23 (median CV of 0.70), which indicates that there are both temporal and spatial variations in toxicity risks and suggests that peak concentrations may influence the apparent toxicity of pesticide mixtures.

### Species sensitivity distributions

SSDs could be generated for 19 pesticides (Table 4). The 7 pesticides that were excluded had too few toxicity data points in literature and current databases for the select species group (bitertanol, butachlor, epoxiconazole, imazalil, myclobutanil, thiabendazole, triadimenol).

**Table 4:** Results of SSD: median HC_5_ (μg/L) and maximum PAF (%) of pesticides in the study sites

				Max. PAF (%)				
Substance	Type	Species (*n*)	HC_5_ (μg/L)	RMD-S	CA-S	RMD-F	CPama-J	URMD-CPama
Azoxystrobin	F	Full (12)	43.7	0.00	0.02	0.02	0.01	0.01
Chlorothalonil	F	Full (41)	6.28	0.00	n/a	0.00	0.00	0.00
Difenoconazole	F	Full (7)	100.9	n/a	n/a	n/a	n/a	n/a
Metalaxyl	F	Full (15)	39,363	nd	n/a	n/a	n/a	n/a
Propiconazole	F	Full (29^a^)	386.9	nd	n/a	n/a	nd	n/a
Pyrimethanil	F	Full (7)	2656	n/a	n/a	n/a	n/a	n/a
Tebuconazole	F	Full (9)	848.1	nd	n/a	n/a	n/a	n/a
Ametryn	H	Full (8)	0.23	**4.48**	**67.44**	**27.62**	**7.61**	**14.22**
Bromacil	H	Full (6)	3.78	nd	0.00	**1.10**	nd	nd
Diuron^a^	H	Full (35^a^)	2.62	0.00	**46.52**	**2.07**	0.14	0.40
Hexazinone	H	Full (7)	6.10	nd	0.00	0.01	nd	nd
Oxyfluorfen	H	Full (11)	0.52	nd	nd	nd	0.94	0.72
Terbutryn	H	Full (17)	5.41	nd	0.01	0.00	nd	nd
Carbaryl	I	Insects (155)	1.58	nd	**6.44**	**3.67**	nd	**2.75**
Chlorpyrifos	I	Fish (159)	6.94	nd	0.01	0.00	0.00	0.00
Diazinon	I	Crustaceans (92)	0.23	nd	**11.47**	**7.20**	**3.96**	**2.20**
Ethoprophos	I	Full (14)	3.12	0.37	**3.11**	**1.83**	0.67	**1.54**
Fenamiphos	I	Full (7)	0.82	0.03	0.17	0.33	0.57	0.47
Terbufos	I	Full (11)	0.10	nd	nd	nd	**2.78**	nd

Moderate risk (PAF > 1 %) in bold text. Full indicates that all species were modeled for the pesticide: primary producers, fish and arthropods for fungicides; primary producers for herbicides; fish and arthropods for insecticides

*nd* no detection, *n/a* unquantifiable (near-zero)

^a^Removed outlier(s)

The pesticides that had the highest toxicity risks were the insecticides carbaryl, diazinon, ethoprophos, and terbufos, which were found to pose moderate (>1 % PAF) or high (>5 % PAF) risks to fish and arthropod species, and the herbicides ametryn, bromacil, and diuron that were found to pose moderate or high risks to primary producers. Each of the assessed fungicides posed only low risks (<1 % PAF) to primary producers, fish, and arthropods. The highest risks of single substances were observed at CA-S, where peak concentrations of the herbicides ametryn and diuron were predicted to affect 67 and 46.5 % of primary producers, respectively, and the insecticide diazinon was predicted to affect 11.5 % of crustaceans at levels higher than 50 % effect (Table 4).

### Multi-substance PAF

**Fig. 2:**
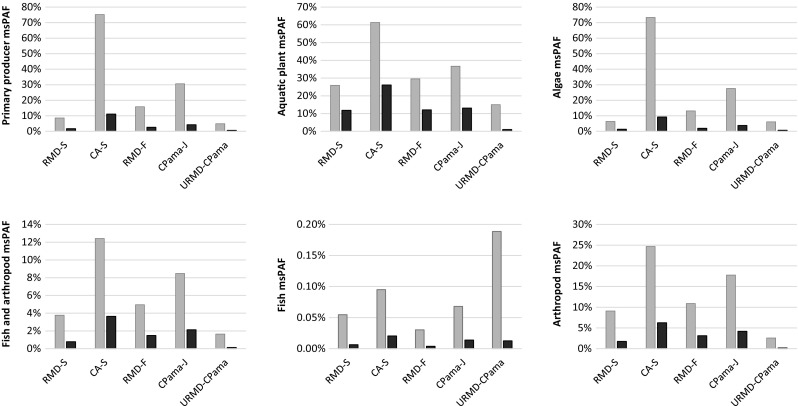
Result of msPAF for six species groups. Maximum msPAF (*gray bars*) and average msPAF (*black bars*) at each site. Note the differences in scale of the *y*-axis between the graphs

The 26 pesticides detected in the field were divided into 19 principal TMoA by chemical groups. Pesticides were further separated into distinct TMoA when SSD slopes differed between pesticides in a TMoA (Table 5). Toxicity data was generally sufficient to include at least 10 pesticides for msPAF assessment on each species group, except for aquatic plants, for which only 2 pesticides could be included (Table 6). We found a moderate to high risk of toxic effects on primary producers (mean 4.0 % msPAF, 9.6 % s.d.) with a peak effect on 75 % of the primary producers at CA-S (Fig. 2). Effects were similar on algae (mean 3.6 %, 9.2 % s.d., max 73 %), whereas there was a higher average effect (mean 12.8 %, 13 % s.d.) but lower peak effect (61 % msPAF) on aquatic plants. The results showed a moderate to low risk (mean 1.6 % msPAF, 2.6 % s.d.) to fish and arthropods, with high risk at peak effect (12 % max msPAF). The risks to arthropods was moderate to high (mean 3.1 %, 4.8 % s.d., maximum 25 %), but the risks to fish was consistently low (maximum 0.2 % msPAF [Fig. 2]).

**Table 5:** The type, chemical group, and TMoA assigned to pesticides assessed in msPAF. Letters (A–F) indicate pesticides placed in distinct TMoA. Pesticides without an assigned TMoA for a species group were not assessed

			Toxic mode of action						
Pesticide	Type	Chemical group	Fish and arthropods	Fish	Fish (N/LOEC)	Arthropods	Primary producers	Algae	Aquatic plants
Metalaxyl	F	Acylamino acid, anilide	1	1	–	1	1	1	–
Pyrimethanil	F	Anilinopyrimidine	2	2	–	2	2	2	–
Chlorothalonil	F	Aromatic	3	3	3	3	3	3	3
Thiabendazole	F	Benzimidazole, thiazole	4	4	–	4	–	–	–
Imazalil	F	Conazole (imidazoles)	5	5	–	5	5	5	–
Difenoconazole	F	Conazole (triazoles)	6^A^	6^A^	–	6^A^	6^A^	6^A^	–
Myclobutanil	F	Conazole (triazoles)	6^B^	6^B^	–	6^B^	6^B^	6^B^	–
Propiconazole	F	Conazole (triazoles)	6^C^	6^C^	–	6^C^	6^A^	6^A^	6
Tebuconazole	F	Conazole (triazoles)	6^C^	6^D^	–	6^D^	6^C^	6^C^	–
Triadimenol	F	Conazole (triazoles)	6^B^	6^F^	–	–	6^E^	6^D^	–
Azoxystrobin	F	Methoxyacrylate strobilurin	7	7	–	7	7	7	–
Bitertanol	F	Triazole	9	9	–	9	9	9	–
Butachlor	H	Chloroacetanilide	10	10	10	10	10	10	–
Ametryn	H	Methylthiotriazine	12^A^	12^A^	–	12^A^	12	12	–
Terbutryn	H	Methylthiotriazine	12^B^	12^B^	–	12^B^	12	12	–
Oxyfluorfen	H	Nitrophenyl ether	13	13	–	13	13	13	–
Diuron	H	Phenylurea	14	14	14	14	14	14	14
Hexazinone	H	Triazinone	15	15	15	15	15	15	15
Bromacil	H	Uracil	16	16	–	16	16	16	16
Ethoprophos	I	Aliphatic organothiophosphate	17^A^	17^A^	–	17^A^	–	–	–
Terbufos	I	Aliphatic organothiophosphate	17^B^	17^B^	–	17^B^	17	17	–
Carbaryl	I	Benzofuranyl methylcarbamate	19	19	19	19	19	19	19
Fenamiphos	I	Phosphoramidate	20	20	–	20	–	–	–
Chlorpyrifos	I	Pyridine organothiophosphate	21	21	21	21	21	21	–
Diazinon	I	Pyrimidine organothiophosphate	22	22	22	22	22	22	–

The fungicide epoxiconazole was excluded from the assessment as there was insufficient toxicity data to assess the substance with any species groups

*F* fungicide, *H* herbicide, *I* insecticide

**Table 6:** Data available for msPAF calculations. Species count (*n*) with data point count (*n*, in parenthesis) and standard deviation (σ) for each detected pesticide

	Fish and arthropods		Fish		Fish (N/LOEC)		Arthropods		Primary producers		Algae		Aquatic plants	
Pesticide	Count	σ	Count	σ	Count	σ	Count	σ	Count	σ	Count	σ	Count	σ
Metalaxyl	6 (18)	0.52	5 (9)	0.37	– (–)	–	1 (9)	0.66	9 (13)	0.69	8 (12)	0.72	1 (1)	–
Pyrimethanil	4 (7)	0.45	3 (4)	0.28	– (–)	–	1 (3)	0.05	5 (7)	0.65	4 (6)	0.65	1 (1)	–
Chlorothalonil	21 (77)	0.45	14 (57)	0.38	2 (3)	0.668	7 (20)	0.59	19 (34)	1.03	10 (15)	0.87	9 (19)	1.14
Thiabendazole	3 (22)	0.69	2 (15)	0.57	– (–)	–	1 (7)	0.32	–	–	–	–	–	–
Imazalil	3 (5)	0.19	2 (3)	0.22	– (–)	–	1 (2)	0.003	2 (3)	0.12	2 (3)	0.12	–	–
Difenoconazole	5 (10)	1.41	4 (8)	1.07	– (–)	–	1 (2)	2.41	3 (3)	1.24	3 (3)	1.24	–	–
Myclobutanil	3 (5)	0.35	2 (3)	0.18	– (–)	–	1 (2)	0.13	3 (4)	0.40	3 (4)	0.40	–	–
Propiconazole	15 (45)	0.60	8 (26)	0.64	– (–)	–	7 (19)	0.54	17 (29)	0.92	15 (24)	0.98	2 (5)	0.61
Tebuconazole	7 (14)	0.54	5 (10)	0.42	– (–)	–	2 (4)	0.32	2 (4)	0.15	2 (4)	0.15	–	–
Triadimenol	4 (5)	0.38	3 (4)	0.10	– (–)	–	1 (1)	–	3 (5)	0.81	3 (5)	0.81	–	–
Azoxystrobin	8 (19)	0.45	4 (5)	0.32	– (–)	–	4 (14)	0.44	5 (10)	1.10	4 (9)	1.12	1 (1)	–
Bitertanol	3 (7)	0.21	2 (4)	0.11	– (–)	–	1 (3)	0.11	1 (2)	0.48	1 (2)	0.48	–	–
Butachlor	17 (28)	0.55	11 (21)	0.42	1 (4)	0.174	6 (7)	0.59	5 (11)	1.77	5 (11)	1.77	–	–
Ametryn	10 (21)	0.45	9 (18)	0.38	– (–)	–	1 (3)	0.21	9 (12)	0.93	9 (12)	0.93	–	–
Terbutryn	8 (21)	0.64	6 (15)	0.29	– (–)	–	2 (6)	0.80	18 (34)	1.02	18 (34)	1.02	–	–
Oxyfluorfen	4 (7)	0.39	3 (5)	0.15	– (–)	–	1 (2)	0.90	13 (17)	1.92	12 (16)	1.92	1 (1)	–
Diuron	28 (121)	0.53	14 (85)	0.47	1 (2)	0.093	14 (36)	0.61	41 (72)	1.11	37 (66)	0.94	4 (6)	1.92
Hexazinone	12 (59)	0.45	10 (51)	0.35	4 (4)	2.248	2 (8)	0.43	6 (18)	0.63	3 (5)	0.57	3 (13)	0.41
Bromacil	5 (11)	0.35	3 (6)	0.28	– (–)	–	2 (5)	0.37	6 (8)	0.43	4 (4)	0.63	2 (4)	0.07
Ethoprophos	14 (71)	0.93	9 (37)	0.73	– (–)	–	5 (34)	1.11	–	–	–	–	–	–
Terbufos	12 (29)	1.28	5 (20)	0.87	– (–)	–	7 (9)	1.70	3 (4)	0.81	3 (4)	0.81	–	–
Carbaryl	157 (661)	1.19	69 (400)	0.48	2 (24)	0.366	88 (261)	1.11	12 (19)	0.58	10 (17)	0.58	2 (2)	0.33
Fenamiphos	7 (21)	0.72	2 (7)	0.73	– (–)	–	5 (14)	0.36	1 (1)	–	1 (1)	–	–	–
Chlorpyrifos	161 (860)	1.34	37 (180)	0.93	5 (15)	0.992	124 (680)	1.26	7 (10)	0.68	7 (10)	0.68	–	–
Diazinon	94 (406)	1.56	53 (222)	0.80	8 (46)	0.531	41 (184)	0.96	9 (12)	0.35	9 (12)	0.35	–	–
Median	7.5 (21)	–	5 (12.5)	–	2 (4)	–	2 (7)	–	6 (10.5)	–	4.5 (9.5)	–	2 (4)	–

Following the low toxicity risk to fish, we produced an msPAF using available data on No and Lowest Observed Effect Concentration (NOEC, LOEC) to assess “onset effects” on fish (effects on a small percentage of fish populations). Sufficient literature NOEC or LOEC data was available for only 3 of 26 pesticides divided into separate TMoA: these were chlorpyrifos, diazinon, and hexazinone. This msPAF on NOEC and LOEC data showed a low average risk to fish (mean 0.1 %, 0.3 % s.d.), with a peak effect causing moderate risk (1.7 % msPAF) at CA-S.

### Determining which pesticides cause highest risks in msPAF

An assessment of the pesticides causing the highest risks in the RMD was conducted by first calculating the cumulative risk posed by each TMoA over the study period as the sum(msPAF_CA_) for each TMoA and species group. The contribution from each pesticide to the cumulative risk of its TMoA was then calculated as the cumulative MEC of the pesticide over the study period, expressed as the sum(MEC) for the pesticide in non-dimensional HU units. This was followed by assigning a fraction of the cumulative risk of a TMoA to each pesticide corresponding to its fraction of cumulative MEC for its TMoA. The result is the fraction of risk contributed by each pesticide to each species group over the study period. The use of cumulative risk is an attempt to describe the relative risks of pesticides without selecting a parameter such as mean or maximum concentrations which could introduce biases stemming from exposure patterns. A pesticide in this system may be ranked among the top risk contributors by posing a frequent but low risk to the environment or by posing an infrequent but high risk. The top ranked pesticides are likely to be those that could be placed into both categories.

This revealed that the herbicides ametryn and diuron and the fungicide difenoconazole are responsible for more than 90 % of the cumulative toxicity risk in primary producers, algae, and aquatic plants (Table 7). Diuron poses a much higher cumulative risk to aquatic plants than to algae: the sum(msPAF_CA_) of diuron is 961 % for aquatic plants and 57 % for algae. This suggested that aquatic plants are much more sensitive to diuron than algae, given that the exposure to diuron is the same for both groups. Similarly, more than 90 % of cumulative risk to fish, arthropods, and the fish and arthropod group is caused by the insecticides: chlorpyrifos, diazinon, ethoprophos, and the fungicide difenoconazole, as well as the insecticide carbaryl for arthropods. The msPAF on fish NOEC and LOEC data suggested that the herbicide hexazinone may cause onset lethal effects in fish, but this herbicide does not contribute to toxicity risks to fish at median effect levels (Table 7).

**Table 7:** Contribution of each pesticide to total cumulative risk in msPAF

Type	Pesticide name	Primary producers (%)	Algae (%)	Aquatic plants (%)	Fish and arthropods (%)	Arthropods (%)	Fish (%)	Fish (N(L)OEC) (%)
Fungicides	Metalaxyl	0.0	0.0	–	0.0	–	0.0	–
	Pyrimethanil	0.0	0.0	–	0.0	–	–	–
	Chlorothalonil	0.1	0.0	0.1	0.0	0.0	0.0	–
	Thiabendazole	–	–	–	–	–	–	–
	Imazalil	–	–	–	–	–	–	–
	Difenoconazole	**5.9**	**9.1**	–	**6.8**	–	**67.8**	–
	Epoxiconazole	–	–	–	–	–	–	–
	Myclobutanil	–	–	–	0.0	–	–	–
	Propiconazole	0.0	0.1	–	0.0	0.0	0.0	–
	Tebuconazole	–	–	–	0.0	–	0.0	–
	Triadimenol	–	–	–	0.0	–	–	–
	Azoxystrobin	3.2	6.8	–	0.0	0.0	0.0	–
	Bitertanol	–	–	–	–	–	–	–
Herbicides	Butachlor	0.	0.2	–	0.0	0.0	0.0	–
	Ametryn	**52.8**	**60.2**	–	0.0	–	0.0	–
	Terbutryn	0.0	0.1	–	0.0	–	0.0	–
	Oxyfluorfen	1.4	1.3	–	0.0	–	–	–
	Diuron	**36.2**	**20.7**	**99.9**	0.0	0.0	0.0	–
	Hexazinone	0.0	–	–	0.0	–	0.0	**96.7**
	Bromacil	0.1	1.4	–	0.0	–	–	–
Insecticides	Ethoprophos	–	–	–	**9.4**	**36.0**	**16.1**	–
	Terbufos	–	–	–	0.7	2.5	0.3	–
	Carbaryl	0.0	0.0	–	4.3	**7.8**	0.0	–
	Fenamiphos	–	–	–	–	0.0	–	–
	Chlorpyrifos	0.0	0.0	–	**48.3**	**35.3**	**15.7**	3.3
	Diazinon	0.0	0.0	–	**30.5**	**18.4**	0.0	0.0

Bold values are the pesticides associated with at least 90 % of cumulative risk to the species group

### Ranking of study sites based on risks

A ranking of the relative risks at the five study sites in the RMD was created using toxicity risk values from the three risk assessment models. Each site was assigned a rank based on relative risk and given a score of 1 for the lowest risk, up to a score of 5 for the highest risk. Sites were ranked based on the average probability of clear effects in PERPEST, the maximum PAF in SSD, and the average msPAF. As several endpoints or species groups were assessed in each model, a total of 15 results were ranked (Table 8). The lowest toxicity risk (score of 22) was found at RMD-S, which is located upstream of most plantation effluents (Fig. 1). The CA-S site located in the Caño Azul tributary had the highest risk (score of 72). Located downstream of RMD-S and CA-S, RMD-F had the second highest risk (score of 49). The second tributary site, CPama-J, ranked third (score of 44), and the URMD-CPama site downstream of CPama-J and RMD-F ranked fourth (score of 38). These results suggest that the largest source of pesticide pollution is the Caño Azul tributary, which initially affects CA-S followed by a gradually declining toxicity risk at the sites further downstream. Thus, the relative risks in the five sites in the RMD watershed are as follows (Table 8):

$$ CA-S>RMD-F>CPama-J>URMD-CPama>RMD-S $$

**Table 8:** Ranking of sites by the relative risks in the three applied models

	Assessed group	RMD-S	CA-S	RMD-F	CPama-J	URMD-CPama
PERPEST	Algae and macrophytes	1	5	4	2	3
	Zooplankton	1	5	3	2	4
	Macroinvertebrates	1	5	4	2	3
	Fish and tadpoles	1	3	4	2	5
	Community metabolism	1	5	4	2	3
SSD	Fungicides	1	4	5	2	3
	Herbicides	1	5	4	2	3
	Insecticides	1	5	4	3	2
msPAF	Primary producers	2	5	3	4	1
	Algae	2	5	3	4	1
	Aquatic plants	2	5	3	4	1
	Fish and arthropods	2	5	3	4	1
	Arthropods	2	5	3	4	1
	Fish	2	5	1	4	3
	Fish (N/LOEC)	2	5	1	3	4
	Total score (avg.)	22 (1.5)	72 (4.8)	49 (3.3)	44 (2.9)	38 (2.5)

Higher values indicate higher toxicity risks

## Discussion

The three risk assessment models showed evident toxicity risks to aquatic organisms due to pesticide pollution in the RMD. The Caño Azul tributary (CA-S), which receives agricultural surface water runoff from pineapple and banana plantations, poses particularly high risks to aquatic organisms and the CA-S site is associated with the highest risks in each of the three models: i.e., a 95 % probability of clear effects on community metabolism in PERPEST, a 67 % PAF for primary producers from ametryn in SSD, and a 75 % msPAF on primary producers. Similarities between the pesticide residue profiles of samples taken at CA-S and the downstream sites RMD-F and URMD-CPama within specific sampling dates show that pesticides travel downstream from the Caño Azul tributary to pollute large areas of the RMD main stem.

We identified 3 pesticides in msPAF associated with 90 % of median toxicity risks to primary producers and 5 pesticides with the same magnitude of effects on fish and arthropods: the fungicide difenoconazole, the herbicides ametryn and diuron, and the insecticides carbaryl, chlorpyrifos, diazinon, and ethoprophos (Table 7). A previous toxicity assessment of pesticide usage in Costa Rica found that 75 % of aquatic ecotoxicity was likely to be caused by diazinon, mancozeb, chlorothalonil, terbuthylazine, and ethoprophos (Humbert et al. 2007). The insecticides chlorpyrifos, diazinon, and ethoprophos were thus some of the most toxic pesticides for aquatic organisms in both the previous and the present study. The present study also found high risks of the fungicide difenoconazole and the herbicides ametryn and diuron which have previously not been reported. Humbert et al. (2007) found a high toxicity risk of the fungicide mancozeb, one of the most commonly used fungicides in this area. Mancozeb was, however, not analyzed in the present study. Many current-use pesticides are still a challenge to detect in environmental samples or not yet analyzed in the pesticide analysis laboratory at IRET. The list of omitted pesticides includes 9 of the 16 most imported pesticides, the majority of which are classified as highly toxic to aquatic biota (De la Cruz et al. 2014). Thus, the actual toxicity risks to aquatic organisms may be underestimated in this study.

### Pesticide pollution and fish kills

Fish kills have been frequently observed in the RMD, and pesticide pollution has been suggested as a probable cause (CGR 2013; Diepens et al. 2014). The present study found that toxicity risks to fish was low in the three risk assessment models. There was a low probability of clear effects on fish and tadpoles in PERPEST; however, the risks to this endpoint are difficult to assess with PERPEST as the underlying case base still contains relatively few fish and tadpole data from mesocosm experiments (1.3 analogous cases, on average) compared to other taxonomic groups (4.2 to 11.4 analogous cases, see Table 3).

The SSD model found high risks to fish and arthropods. However, the fish and arthropod group was found in msPAF to underestimate risk to arthropods and to overestimate risks to fish (compare in Fig. 2), suggesting that results from the fish and arthropod SSDs cannot be used to predict effects on either fish or arthropods separately. This is also seen in the SSD results, where fish were more robust than arthropods in three cases where SSDs were derived for fish and arthropods as separate groups: a fish SSD was reported for chlorpyrifos only because the arthropod toxicity data was non-log-normal distributed (Table 4). Additionally, there was a low risk to fish in msPAF (<1 % msPAF), which suggests that large mortality events (>50 % mortality in a fraction of species) are not likely to occur from exposure to the pesticide mixtures measured in the RMD during the 2-year sampling period.

There was a moderate risk to fish (>1 % msPAF) in the msPAF using NOEC and LOEC data, but these effect levels are widely acknowledged to be of poor quality (Jager 2012; Landis and Chapman 2011; Laskowski 1995). Although these effect levels aim to describe the highest concentration not causing (or the lowest concentration causing) toxic effects in organisms, they are associated with other toxic effect levels in practice, see Crane and Newman (2000). Nevertheless, the three pesticides assessed were found to cause a low to moderate risks of such “onset effects.”

Overall, the three risk assessment models suggest that fish are less affected than other taxonomic groups and the detected concentrations of pesticides cannot explain the observed mass mortality of fish in the RMD. The apparent risk of pesticides may, however, be underestimated as highly toxic insecticides such as pyrethroids were not analyzed in this study, and because pesticide concentrations are expected to peak following pesticide application or heavy rainfall, while our sampling efforts did not aim to measure such peak concentrations. However, if we define peak concentrations as statistical outliers (>2 standard deviations [σ] above the mean), we did observe peak concentrations for 12 out of 26 pesticides. Two herbicides had particularly high peaks at 5.9 σ (ametryn) and 6.2 σ (diuron) above their means. Some insecticides reported in the present study are applied at high doses a few times per year to control nematode pests, but the measured peaks of insecticides were lower than those of herbicides (chlorpyrifos [3.5 σ], diazinon [3.6 σ], ethoprophos [4.2 σ]). We also acknowledge that it is possible that pesticide concentrations and associated risks to aquatic organisms are occasionally higher than those reported in the present study as the number of samples (15 per site) may be considered small in relation to the high variability observed in pesticide occurrence. Given that we did not observe fish kills during the sampling effort, unobserved peaks of pesticides (particularly insecticides used as nematicides) remain a potential cause that merits further investigation. We recommend further investigation into the toxicity risks of short pesticide pulses (e.g., peak concentrations) associated with pesticide application and rainfall.

An investigation in the Sixaola River on the Caribbean coast of Costa Rica assessed the toxicity of three pesticides (chlorpyrifos, difenoconazole, and terbufos) in water samples and passive samplers already deployed during fish kill events using hazard quotients and found that measured pesticide concentrations did not pose a high risk of mortality in fish (Polidoro and Morra 2016). They concluded that a combination of multiple stressors, e.g., mixtures of pesticides, low oxygen content, high temperatures, high nutrients, and ecological effects including species interactions, may contribute to lower toxicity risk thresholds in the Sixaola watershed. The present study investigated the effects of pesticide mixtures, as well as ecological effects of pesticides, using the PERPEST model and found that clear effects on fish and tadpoles were still unlikely. Similarities and differences between the Sixaola study and the present study in the RMD show that toxicity studies need to assess a wider range of pesticides and include other stressors to gain a better understanding of the most probable causes of mortality in fish populations in tropical aquatic ecosystems. Stressors are often coupled, for example, heavy rainfall leads to runoff of fertilizers and soil in addition to pesticides, which may cause eutrophication and oxygen depletion. Eutrophication may be further associated with harmful algae blooms that cause fish kills (Paerl and Otten 2013) and have been documented on the Pacific coast of Costa Rica (Vargas-Montero et al. 2006). Algae blooms have not yet been reported or studied in the RMD, but the presence of herbicides may provide a competitive advantage for blooming cyanobacteria (Lurling and Roessink 2006) and decomposing blooms may contribute to oxygen depletion and fish kills in the RMD (Diepens et al. 2014).

### Comparison and recommended use of the models

The three risk assessment models used in this study are all based on comparing environmental concentrations to toxicity benchmark values obtained from the literature. The models apply different methods to assess risks, and the similarities and dissimilarities, and strengths and weaknesses, of each model are discussed below for the purpose of future ERA use in similar aquatic ecosystems.

The PERPEST model uses data gathered from mesocosm experiments (where pesticides are applied in ponds or tanks containing organisms from several trophic levels, e.g., microalgae, macrophytes, zooplankton, benthic invertebrates, fish). Apart from measuring direct effects (e.g., mortality) on multiple species, mesocosms allow assessment of indirect ecological effects (e.g., prey-predator interactions) of single pesticides or pesticide mixtures. This means that PERPEST has the highest ecological relevance out of the three models, but the absence of established threshold risk values (such as HC_5_ in SSD) makes the PERPEST model challenging to use in risk management. However, threshold values of acceptable probabilities could be set easily (e.g., 10 % probability of clear effect). Additionally, few mesocosm toxicity data available for fish and tadpoles may pose a problem in risk assessment, as fewer analogous cases lead to higher uncertainty in fish and tadpoles than other endpoints. On the other hand, the relative ease of meeting data requirements, the wide range of assessed endpoints, and high ecological relevance of the PERPEST model make it ideal for screening risk assessments of pesticide mixtures occurring in the field. It is also a more comprehensive approach than the risk (or hazard) quotient approaches currently used for screening risk assessments in Costa Rica, see, e.g., Polidoro and Morra (2016).

The SSD model uses data from single species toxicity tests and is dependent on data availability for its accuracy. There are many views on data requirements for SSDs, but the confidence interval (CI) is sometimes used to specify the uncertainty of SSD predictions. This has been shown to be a fixed number depending on the sample size of data in a normal distribution: at a 5 % PAF, *n* = 3 results in a 46 % upper confidence limit, decreasing to 20 % at *n* = 10, and 12 % at *n* = 30 (Aldenberg and Jaworska 2000). Wheeler et al. (2002) similarly observed that their SSD outputs stabilized at 10–15 data points and consequently recommended that regulatory decisions should be based on SSDs of at least 10 species. The present study used a pragmatic approach to allow assessment of 19 of 26 pesticides with a minimum of 6 species, but 12 pesticides could be modeled using at least 10 species (Table 4). The present study used a moderately wide selection of species for SSDs and assessed primary producers separately from fish and arthropods. However, these SSDs describe the sensitivity of the collective species group and may leave smaller, important groups of species at risk despite these being assessed. We have shown that assessing toxicity data on fish and arthropods together is overprotective for fish and underprotective for arthropods based on msPAF (see Fig. 2). Species selection consequently has a large impact on the interpretation of results when extrapolating or interpolating effects to other species than the precise group that is assessed. Similar effects have been observed for herbicides, and only the most sensitive primary producers were recommended to be used in SSDs (Van den Brink et al. 2006a). Our results also show that three or four pesticides are required to explain 90 % of cumulative mixture toxicity in well-studied species groups in the RMD (Table 7). This suggests that risks derived for individual substances are poor estimates for mixtures and consequently that SSDs are not suitable for assessing mixtures. The SSD concept has an established role in setting environmental threshold concentrations (i.e., EQS) for individual toxic substances, but the present study show that mixture scenarios should be considered when deriving EQS for highly polluted ecosystems. Mixture assessment factors have been proposed as a method to account for mixture effects when developing EQS, see Backhaus et al. (2010).

The msPAF model uses the principles of the SSD concept to assess the toxicity of complex mixtures. We found that msPAF resulted in higher risk values than those predicted by the single substance approach of SSDs, and that the toxicity of mixtures should be considered over single substances when pesticide mixtures occur in the field. However, further research is needed to determine which available TMoA classifications are the most suitable for use in msPAF and other toxicity models (such as PERPEST) that aim to apply both CA and RA models to assess the toxicity of mixtures.

**Fig. 3:**
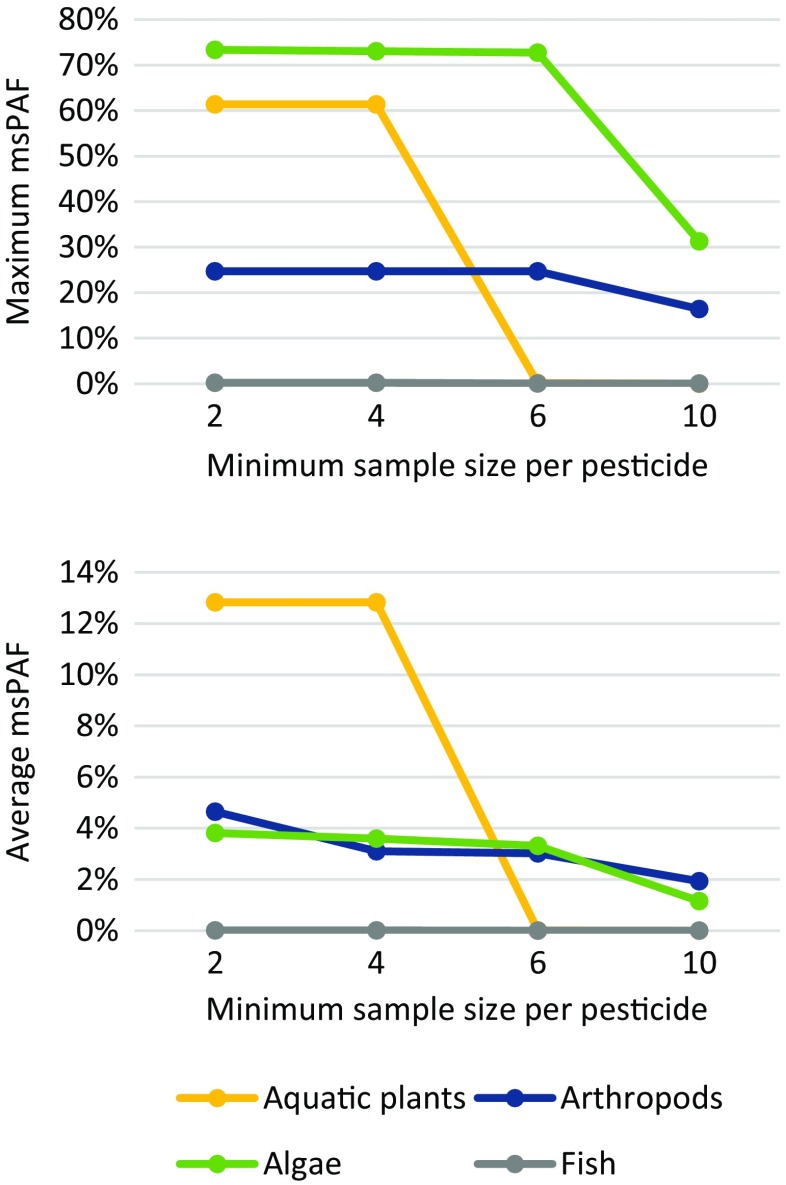
The maximum and average msPAF toxicity values for four species groups in the RMD at four select minimum sample sizes

**Fig. 4:**
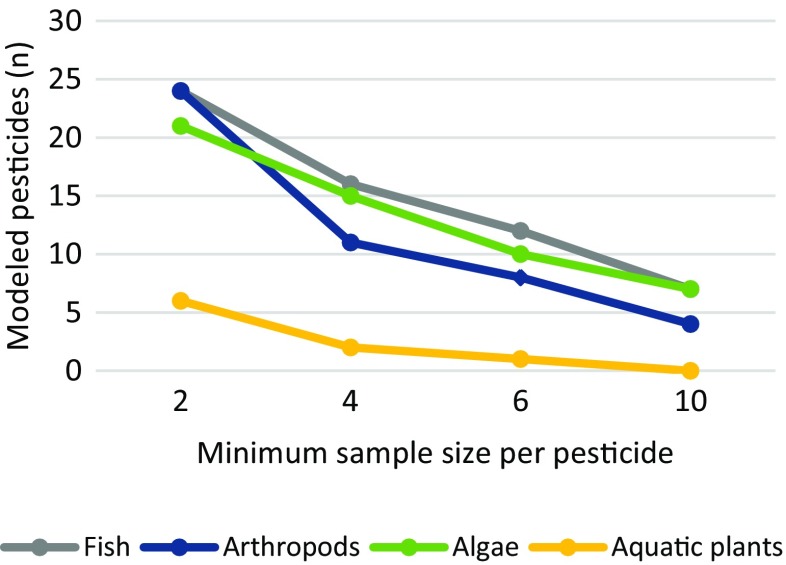
Number of pesticides (*n*) available for msPAF modeling of four species groups in the RMD at four select minimum sample sizes

The present study has found that setting a minimum sample size for msPAF may have negative effects on the results of a mixture risk assessment. We compared the msPAF results in the present study over a range of hypothetical minimum sample sizes (2, 4, 6, and 10 species) and found that the apparent toxicity (as an estimate of actual toxicity) to species with scarce data (aquatic plants) diminished above a 4 species minimum, and that the apparent maximum toxicity to a well-studied species group (algae) was strongly reduced above a 6-species minimum (Fig. 3). Furthermore, the number of assessed pesticides decreased continuously with an increase in minimum sample size (Fig. 4). These trends suggest that implementing a minimum number of species (or toxicity tests) may lead to a less protective risk assessment, as the excluded fraction of mixture components may strongly contribute to the apparent toxicity of the mixture. The msPAF model may, as part of a first tier assessment, be used to support a decision between further quantification of risks, remediation actions, or approval of the ecological status of an ecosystem, and it is therefore paramount that risks are not underestimated due to an assessment being carried out on partial mixtures.

### Conclusions

The present study has found that pesticides detected downstream banana, pineapple, and rice plantations in the River Madre de Dios (RMD) pose high risks to zooplankton, macroinvertebrates, algae, macrophytes, and overall community metabolism. Measures are urgently needed in order to reduce these toxicity risks and the release of highly toxic pesticides into the RMD. Seven pesticides were identified to cause 90 % of apparent toxicity risks in the msPAF model: the fungicide difenoconazole, the herbicides ametryn and diuron, and the insecticides carbaryl, chlorpyrifos, diazinon, and ethoprophos.

This study included 26 pesticides that could be analyzed and were detected in the RMD, but several other pesticides are applied in these plantations, such as mancozeb and pyrethroids, that were not included as they are more difficult to analyze in the local laboratories. The apparent toxicity risks may therefore be underestimated, further stressing the need for mitigation actions in the RMD. We suggest that further studies should be carried out to determine the causes of reported fish kills, focusing on peak concentrations following pesticide application and rainfalls and multiple stressors other than pesticides (e.g., nutrients, oxygen content, temperature, algal blooms). The PERPEST model was found to be well-suited for screening risk assessments of pesticide mixtures. The SSD concept can be used to set protective environmental quality standards for single substances within mixtures provided appropriate safety factors are used. The msPAF model was here found to be the most comprehensive tool for environmental risk assessment of mixtures and offers the advantage of assessing pesticides with very limited toxicity data provided that their toxic modes of action are known.
